# Supplementary material for: Long-term drift of Si-MOS quantum dots with intentional donor implants
Source: Sci Rep. 2019 May 21;9:7656. doi: 10.1038/s41598-019-43995-w (PMC6529408; doi:10.1038/s41598-019-43995-w)
Supplement: Supplementary file 1 — Supplementary Information [file 41598_2019_43995_MOESM1_ESM.pdf]

Long-term drift of Si-MOS quantum dots with intentional donor implants

M. Rudolph,[1] B. Sarabi,[2] R. Murray,[2] M.S. Carroll,[1] and Neil M. Zimmerman[2]

[1]*Sandia National Laboratories, Albuquerque, NM, 87185, USA*

[2]*National Institute of Standards and Technology, Gaithersburg, MD, 20899, USA*

Supplemental Information for Energy vs. Charge Units

Previous measurements of charge noise have been reported both in units of energy (where the noise is extracted from qubit dephasing)[1,2] and charge (where the noise is directly extracted from fluctuations of the current passing through a QD)[3]. In the context of qubit dephasing, the charge noise is best denoted in units of energy, which directly describes the fluctuations of the QD energy levels induced by nearby charge noise. Experiments measuring the charge noise by qubit dephasing typically observe quasi-static values from 1-10 μeV[1,2]. In addition to qubit gate fidelity, low-frequency charge noise can also affect the qubit readout fidelity. To read out the qubit state, many schemes utilize a single electron transistor (SET) to detect the charge state of the QD, which can infer the qubit state[4]. Low-frequency charge noise can modify the readout sensitivity and require frequent calibrations of the read-out circuit. The maximum sensitivity of the SET is often constrained by the limiting energy scale in SET (i.e. the electron temperature or bias voltage), and is typically < 100 μeV. This scale sets a maximum amount of low frequency drift that can be tolerated before the read-out circuit needs to be retuned. For this application, describing the charge noise in units of energy allows direct comparison to the energy scales of the read-out circuit. The same is true for devices that use a quantum point contact for the read-out electrometer[5].

On the other hand, the low-frequency charge noise has been described by the charge offset drift parameter, which extracts the capacitively-coupled displacement charge induced on the SET by the environmental charge noise[3,6]. This quantity is appropriate when discussing integrating classical or quantum devices. It is also appropriate when the read-out SET is tuned such that the Coulomb blockade is quasi-sinusoidal, which happens when $k_B T \sim E_C/5$ or when the SET tunnel barriers are tuned to be open. Here E_C is the SET charging energy. Previous measurements operated in this regime.

The charge offset drift and chemical potential drift can both be related to the specific device SET and disorder geometry[7]

$$Q_0(t) = -e \sum_i \frac{C_{m,i}(t)}{C_{d,i}(t)}, \tag{1}$$

$$\mu_0(t) = \frac{e^2}{C_\Sigma} \sum_i \frac{C_{m,i}(t)}{C_{d,i}(t)}, \tag{2}$$

where C_m is the mutual capacitance of a fluctuating charge i, C_d is the total capacitance of the disorder charge, and $C_\Sigma = e^2/E_C$ is the total capacitance of the SET. The sums are over a set of charged defects where each defect has a charge magnitude of $|e|$. The motion in the defect charge, which leads to time dependence in the capacitances C_m and C_d. In general, C_Σ can also have a time dependence, but for the specific case where the single electron transistor (SET) is much larger than the disorder feature, as is valid here, the time dependence is negligible.

To gain more insight for implications of measuring charge offset drift and chemical potential drift, we consider the effect of a single defect by the capacitance network described in Fig. 1. The total capacitances for the SET and disorder site are $C_\Sigma = C'_\Sigma + \frac{C_m C'_d}{C_m + C'_d}$ and $C_d = C'_d + \frac{C_m C'_\Sigma}{C_m + C'_\Sigma}$, respectively. We consider two limits for the disorder: $C_m/C'_d << 1$ and $C_m/C'_d >> 1$. The first limit describes disorder that is relatively far away from the SET while the second limit describes disorder that is close to the SET. Expanded in orders of C_m, the contribution to the total

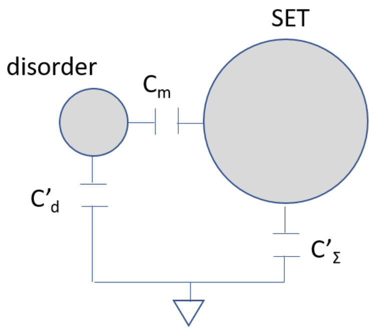

FIG. 1. Capacitance network for a SET and disorder site.

charge offset and SET chemical potential of disoder site i are

$$Q_{0,i} = -e\frac{C_m}{C'_d}\left[1 - \frac{C_m}{C'_d}\right], \qquad \tfrac{C_m}{C'_d} << 1 \tag{3}$$

$$Q_{0,i} = -e\left[1 - \frac{C'_d}{C_m} + \frac{C_m}{C'_\Sigma}\right], \qquad \tfrac{C_m}{C'_d} >> 1 \tag{4}$$

$$\mu_{0,i} = e^2\frac{C_m}{C'_d C'_\Sigma}\left[1 - \frac{C_m}{C'_d} + \frac{C_m}{C'_\Sigma}\right], \qquad \tfrac{C_m}{C'_d} << 1 \tag{5}$$

$$\mu_{0,i} = e^2\frac{1}{C'_\Sigma}\left[1 - \frac{C'_d}{C_m} + \frac{C_m}{C'_\Sigma} - \frac{C'_d}{C'_\Sigma}\right], \qquad \tfrac{C_m}{C'_d} >> 1. \tag{6}$$

We consider the impact of comparing different device geometries, which translates to changes in the SET size in this simplified capacitance network. Specifically, different devices would have different C_m and C'_Σ, while C'_d and the ratio C_m/C'_Σ would be relatively insensitive to changes in SET size. Within this model, we find that the charge offset drift is first order insensitive to device differences for $C_m/C'_d >> 1$, while the chemical potential drift is inversely proportional to the SET size. For $C_m/C'_d << 1$, the opposite occurs, with the charge offset drift being sensitive to the SET size while the chemical potential drift is first order insensitive *Thus, given a similar disorder distribution in different devices, comparing the charge offset drift characteristics is an indirect measure of the disorder distribution if the majority of the disorder drift satisfies $C_m/C'_d >> 1$, while comparing the chemical potential drift characteristics relates to the disorder distribution for the majority of the disorder satisfying $C_m/C'_d << 1$.*

There are three basic fluctuator types in a device that contribute to the total drift, and their capacitance contributions $C_m(t)/C_d(t)$ are the following (ignoring interactions between fluctuators):

- an isolated two-level fluctuator:

$$\left[\frac{C_{m,a}}{C_{d,a}} - \frac{C_{m,b}}{C_{d,b}}\right] N_a(t) + \frac{C_{m,b}}{C_{d,b}},$$

  with a and b denoting the two charge levels and $N_a = \{0, 1\}$ is the time-dependent occupancy of level a. This scenario may occupy the entire spectrum of C_m/C'_d.

- a charge fluctuating between a disorder site and an electron reservoir:

$$\frac{C_{m,a}}{C_{d,a}} N_a(t),$$

with a denoting the disorder site. We note that while, in theory, C_m/C_d approaches 1 when the disorder site is very close to the SET, in actual devices this fluctuator has $C_m/C_d < 1/2$ ($C_m/C_d' < 1$). This is due to screening of the disorder charge by the reservoir. This scenario is thus biased towards $C_m/C_d' << 1$.

- a charge fluctuating between a disorder site and the SET:

$$\left[1 - \frac{C_{m,a}}{C_{d,a}}\right] N_a(t),$$

with a denoting the disorder site. In thise case, if $C_m/C_d' >> 1$, then $[1 - C_{m,a}/C_{d,a}] \approx 0$. Thus, this scenario is biased towards $C_m/C_d' \lesssim 1$.

Overall, the limit the disorder satisfies is dependent on the disorder distribution and device geometry, and the preferred use of charge offset drift or chemical potential drift for device comparisons is not universal. A more careful study of the device geometry is required to tackle this problem.

We suggest that expressing the charge offset drift in energy units is particularly useful in the contexts of i) dephasing in qubits and/or ii) read-out with a SET or point contact that has a cusp-like response, where the width of the cusp is set by an energy scale. In addition, units of energy will provide a natural comparison between charge noise and other mechanisms that can be coupled to the qubit system, such as phonons[8]. We suggest that expressing the drift in charge units is useful in discussing integration of multiple qubits, or when a SET does not have a cusp-like response set by an energy scale.

---

[1] O. Dial, M. D. Shulman, S. P. Harvey, H. Bluhm, V. Umansky, and A. Yacoby, Physical review letters **110**, 146804 (2013).

[2] K. Eng, T. D. Ladd, A. Smith, M. G. Borselli, A. A. Kiselev, B. H. Fong, K. S. Holabird, T. M. Hazard, B. Huang, P. W. Deelman, et al., Science advances **1**, e1500214 (2015).

[3] N. M. Zimmerman, W. H. Huber, B. Simonds, E. Hourdakis, A. Fujiwara, Y. Ono, Y. Takahashi, H. Inokawa, M. Furlan, and M. W. Keller, Journal of Applied Physics **104**, 033710 (2008).

[4] L. DiCarlo, H. J. Lynch, A. C. Johnson, L. I. Childress, K. Crockett, C. M. Marcus, M. P. Hanson, and A. C. Gossard, Physical Review Letters **92**, 226801 (2004).

[5] J. Elzerman, R. Hanson, J. Greidanus, L. W. Van Beveren, S. De Franceschi, L. Vandersypen, S. Tarucha, and L. Kouwenhoven, Physical Review B **67**, 161308 (2003).

[6] N. M. Zimmerman, C.-H. Yang, N. S. Lai, W. H. Lim, and A. S. Dzurak, Nanotechnology **25**, 405201 (2014).

[7] W. G. Van der Wiel, S. De Franceschi, J. M. Elzerman, T. Fujisawa, S. Tarucha, and L. P. Kouwenhoven, Reviews of Modern Physics **75**, 1 (2002).

[8] F. Beaudoin and W. A. Coish, Physical Review B **91**, 165432 (2015).
